# Developing disability inclusive coaching principles within community recreational sport programming for children

**DOI:** 10.1016/j.jsampl.2025.100109

**Published:** 2025-07-09

**Authors:** Roxy H. O’Rourke, Tara Joy Knibbe, Amy C. McPherson, Guy E. Faulkner, F. Virginia Wright, Kelly P. Arbour-Nicitopoulos

**Affiliations:** a Faculty of Kinesiology and Physical Education, University of Toronto, 55 Harbord St., Toronto, ON, M5S 2W6, Canada; b Bloorview Research Institute, Holland Bloorview Kids Rehabilitation Hospital, 150 Kilgour Rd., Toronto, ON, M4G 1R8, Canada; c Dalla Lana School of Public Health & Rehabilitation, University of Toronto, 155 College St., Toronto, ON, M5T 3M6, Canada; d School of Kinesiology, University of British Columbia, 6081 University Blvd., Vancouver, BC, V6T 1Z1, Canada

**Keywords:** Inclusive coaching, Family-centered care, Disability sport, Quality participation

## Abstract

**Background:**

Positive sport experiences for children are essential to establish long-term sport engagement. Coaches play a key role in fostering quality sport experiences for all children. For children with disabilities, caregivers are often closely involved in their sport participation, particularly when additional support is needed, and their perspectives can offer valuable insight into sport program experiences. The purpose of this study was to understand the role coaches have in optimizing the participation experiences of children in recreation-level sport programs. Specifically, this study aimed to explore children’s, caregivers', and coaches' perceptions of the coaching role in recreational sport programs that involve children with and without disabilities.

**Methods:**

Individual interviews were conducted with 14 children (seven with disabilities), 15 caregivers, and 12 recreational sport coaches. Interview transcripts were analyzed using inductive thematic analysis.

**Results:**

Four themes were generated during the analysis: (a) creating avenues for connection (two subthemes), (b) an environment focused on child empowerment (two subthemes), (c) autonomy-supportive coaching practices (three subthemes), and (d) flexibility and adapting the program to suit everyone (two subthemes). A family-centered coaching approach that prioritizes developing rapport with caregivers to enhance the child’s participation needs is key, especially in sport settings that include children with diverse abilities.

**Conclusions:**

Coaches should employ autonomy-supportive coaching, supporting child connections without forcing them, and provide each child with opportunities for challenge and success. Doing so can help to foster quality participation experiences for children during this formative time to contribute to lasting sport involvement.

**Key points**

1. This study highlights that, while coaches play a key role in the sport participation of children with and without disabilities, caregivers, who are often closely involved in sport due to their child’s support needs, can provide valuable insight into participation experiences and coach interactions.
2. Four key themes, based on coach, child, and caregiver experiences, were developed: creating avenues for connection, an environment focused on child empowerment, autonomy-supportive coaching practices, and flexibility and adapting the program to suit everyone.
3. To enhance sport participation experiences, coaches should embrace an autonomy-supportive approach, allowing children to interact without forcing social interactions, and offering opportunities for children to both be challenged and experience success.

## 1 Introduction

Nearly half of children and youth between the ages of six and 17 engage in recreational sport [1]. The benefits of sport for children are well-recognized, including enhanced psychosocial development [2], and physical and mental health [3]. Sport sampling, where children engage in ‘deliberate play’ in various sports that they find inherently enjoyable, is one pathway towards long-term participation [4]. As per Cote’s developmental model of sport participation [4], an ideal sport trajectory involves entry into sport at age six, followed by a period of sport sampling (ages seven to 12) that entails deliberate play, and often, facilitates the development of sport expertise [5,6]. This sampling period is theorized to prevent over-involvement and specialization in one sport, reducing the risk of burnout and sport dropout [7,8].

Having the opportunity to sample multiple sport types during childhood is associated with a physically active lifestyle in adulthood [9,10]. This evidence highlights the importance of providing sport programs during the formative sampling years that are developmentally appropriate, safe and inclusive, well-organized (i.e., ‘quality sport’; [11]), and personally satisfying and enjoyable [12]. Such programs help foster positive youth development and lay the foundation for lifelong participation in physical activity and overall health [13,14].

Coaches play a pivotal role in shaping a child’s sport experience during the sampling period, and ultimately facilitate continued motivation for pursuing sport [15]. Several studies have found coaches' interpersonal styles and behaviours to be predictive of participants' engagement, enjoyment, and investment in sport (e.g., [16,17]). Providing quality sport programs for children is often challenged by the varied abilities, interests, and motivational climates of groups [18,19]. Some children may be involved in sport for the social benefits, while others may place more value and effort on task accomplishment and/or goal pursuit [20,21]. Balancing these varied interests, motivations, and goal pursuits often depends on the ability of the coach to use effective strategies to facilitate teamwork, social unity, and shared experiences [19,22,23].

Coaches' abilities to manage the group dynamics of sport programs may be further challenged in group settings that involve children with disabilities [24,25]. While the sport experiences of children with disabilities are not always negative, there are dynamic and complex person-task-environment interactions that need to be considered by coaches when working with groups that involve children with disabilities. For example, studies have reported on teasing, bullying, and victimization that children with disabilities encounter in sport settings (e.g., [26]). There are often complexities of the social relationships formed between children in sport programs for ‘all’ abilities. Children without disabilities may, for example, adopt the role of “helper’ rather than ‘friend’, often feeling the need to assist children with disabilities (e.g., pushing a wheelchair; [24]). Whether instructed to perform these roles or not, this often results in the formation of an obligated, as opposed to, authentic peer relationship [27,28].

Another factor linked to children’s sport participation is the role of caregivers. Specifically, caregivers of children with disabilities are often more involved in sport program selection and administration compared to children without disabilities [29,30]. Reasons vary, but may include wanting to ensure coaches have suitable adaptations in place within the program environment to meet their child’s needs [31], and caregivers' concerns related to inadequate coach training [30], which often results from the challenge of adapting skills to meet children’s individual needs within a group. Skill disparities are more pronounced in groups that involve children with diverse abilities (e.g., children using an assistive device in the same program as children who are ambulatory). Depending on a coach’s skill in navigating these individual abilities, such diverse groups may increase the risk of social evaluation and negative sport experiences [32,33].

In the current study, we aimed to understand the role of coaches in optimizing the participation experiences of children in group sport programs that include children with and without disabilities from the perspectives of children, caregivers, and coaches. Aligning with the developmental model of sport participation [4], sport programs that are recreational in nature are those that primarily focus on enhancing children’s enjoyment and participation.

## 2 Methods

### 2.1 Study design

A qualitative descriptive study design was used [34]. Interviews were conducted with children (with and without disabilities), caregivers, and coaches involved in group sport programs that include children with and without disabilities (hereafter ‘inclusive sport programs’). Interviews were either conducted individually with the participant (n ​= ​28) or in dyads, if preferred. Dyads consisted of a child and their caregiver (five dyads), a child and their sibling (one dyad), or multiple caregivers of the same child (one dyad). Consistent with the developmental model of sport participation, a focus on children in the later stages of sports sampling (i.e., ages 10 to 13) was prioritized during recruitment. Ethics approval was granted by the research ethics board at one university and one rehabilitation centre in Ontario, Canada. Children, caregiver and coach participants all provided informed consent.

### 2.2 Participant recruitment and procedures

Purposive sampling was used to recruit children and their caregivers, along with coaches within a metropolitan region in Ontario, Canada through a therapeutic recreation and life skills program, integrated child and youth recreational physical activity programs, local community centres, and word of mouth referrals. Program staff sent study invitation letters to eligible participants. Study eligibility criteria are outlined in Table 1.

**Table 1:** Eligibility criteria for all study participants.

Participant	Criteria
**Children**^a^	(a) Aged 10–13 years,
	(b) Self-reported to be engaging in less than the recommended 60 ​min per day of moderate to vigorous intensity physical activity ([35]; as a proxy for recreational involvement in sport),
	(c) Able to speak English, and
	(d) Had the expressive and receptive language skills to participate in a 30–60-min interview with a researcher
**Caregivers**	(a) A primary caregiver of a child meeting the eligibility criteria, and
	(b) Able to speak English
**Coaches**^b^	(a) Currently coaching recreational-level sport programs for children aged 10–13 years

∗*Note*. ^a^For those children with a disability, a self- or caregiver-identified diagnosis of a disability was provided (e.g., cerebral palsy, autism). ^b^Both coaches with and without experience coaching children with disabilities were eligible to participate to allow for more diverse coaching perspectives.

Interested families and coaches contacted the research coordinator for further study information followed by eligibility screening of those who were interested in participating. The research coordinator arranged a convenient time to meet with eligible participants to assess cognitive capacity for providing consent (child participants only) and to obtain written informed consent (all participants). Interviews were held in a private room at the rehabilitation centre or university and lasted from 35 to 80 ​minutes. The research coordinator (TJK) used a semi-structured interview guide to conduct each interview. Interviews were individualized to each child in terms of probes that followed the main questions. The interviewer, who was trained in qualitative methods, had experience conducting research with children with disabilities, and worked as a research assistant on several research projects at the partnering rehabilitation centre, used probes such as, “tell me more’, “who was with you”, “tell me how that made you feel" to be able to gather rich data on the contextual factors associated with participants’ experiences in the sport programs they spoke about. All interviews were audio-recorded and transcribed verbatim. Example interview questions are shown in Table 2, and the full semi-structured interview guides for children, caregivers, and coaches are provided in the Supplementary Material.

**Table 2:** Sample interview questions.

Children	Caregivers	Coaches
Have you ever participated in a physical activity program that involved kids of all physical and intellectual abilities?	Would you like your child to have more experiences with inclusive physical activity programs? Why or why not? What do you expect your child to get out of taking part in this kind of program?	Can you tell me about a time that you felt a child was not included in a physical activity program? What did you do to help mitigate this?
Can you tell me about a time that you did not feel included in a physical activity program? What did people do in this program that made it *not* inclusive?	In your opinion, what helps to motivate your child to do physical activity? How do you try to motivate your child to do physical activity?	How would you improve a physical activity program to make it more inclusive?
Can you tell me about the relationships you have developed or would develop in inclusive physical activity programs? With kids or adults?	If there was someone to help your child participate in inclusive physical activity programs, what would their purpose be? Help child achieve goals? Modify activities? Observe and give feedback?	How do you perceive your role in helping children participate in inclusive physical activity programs?
How would you feel about having someone there to help you participate and/or feel included? How important is this to you?	What do you feel the most important characteristics are for a coach to have?	How do you imagine the role of the parent in the ‘coaching’ process?
What would be important for the ‘coach’ to know so they could be most helpful to you?	How would you want your child to interact with their coach?	Do you feel having a coach would change how children interact with each other in inclusive physical activity programs? Why or why not?

### 2.3 Data analysis

Data were analyzed using inductive thematic analysis [36]. While existing theoretical approaches in sport psychology and child development exist and could have been used as a starting point for a deductive analysis (e.g., [4]), these frameworks were not developed within populations with disabilities, and thus an inductive analytical approach to this data was selected. Interviews were transcribed verbatim and checked by the research coordinator to ensure fidelity to the interview. The senior author (KAN) and research coordinator (TJK) read all transcripts and developed an initial coding scheme, which remained flexible throughout the analysis. Data analysis occurred concurrently with data collection. As such, the developing coding scheme, including the discovery of repeated patterns and arising questions or ideas, was used to inform subsequent interviews. NVivo software [37] was used to facilitate the analysis.

After preliminary interviews were transcribed, coded, and categorized, research team members (TJK and KAN) met to discuss additional codes and potential theoretical approaches. Codes were added and data were recategorized and/or renamed as necessary. The initial categories were sorted into candidate themes and subthemes. Candidate themes and subthemes were discussed as a team and refined to ensure data within themes were coherent with each other, and to make distinctions between themes. Themes and their corresponding subthemes were then defined with a detailed analytical summary written for each. Representative verbatim excerpts were input into a table with their corresponding theme and subtheme. An audit trail of all analytical thoughts and decisions was documented throughout all stages of data collection and analysis. Transcripts for coaches, children, and caregivers were analyzed simultaneously and given the same weight during the interpretation of the findings.

### 2.4 Author’s influences and methodological rigor

The authors are primarily from a disability research background, having worked in rehabilitation hospitals and kinesiology faculties at universities. Throughout data analysis, methodological rigor and trustworthiness were evaluated using the criteria of credibility, dependability, confirmability, and transferability [38]. Examples of how each criterion of credibility was fulfilled include iterative data collection and analysis (e.g., interviewing and coding), reasonable interview length (35–80 ​minutes), and equal attention given to data during coding. Dependability was achieved by field note taking, language reflecting the active role of the researchers, and an audit trail for tracking decisions (e.g., theoretical choices). Confirmability was addressed through regular team meetings and comparing themes (e.g., intensive sorting of data excerpts into a table of candidate themes). Our transparency regarding participant information, robust write-up of the analysis with data excerpts, and the interpretive discussion of our analysis facilitate transferability of the results.

## 3 Results

The sample consisted of 14 children (10–13 years; seven with a physical [e.g., cerebral palsy] or neurodevelopmental [e.g., autism] impairment, seven without a disability), 15 caregivers, and 12 recreational sport coaches with (n ​= ​8) and without (n ​= ​4) experience instructing children with a disability in a sport setting. See Tables 3 and 4 for a summary of participant demographic characteristics. The results are organized according to four resulting themes: (a) creating avenues for connection, (b) an environment focused on child empowerment, (c) autonomy-supportive coaching practices, and (d) flexibility and adapting the program to suit everyone. Each theme is defined by its associated sub-themes. Illustrative quotes from participants are provided with pseudonyms to maintain confidentiality.

**Table 3:** Summary of child and caregiver demographic characteristics.

Child Pseudonym	Sex	Functional Mobility Status	Sports	Caregiver Pseudonym	Caregiver Relationship
Anthony	Male	Walks with no help	Gymnastics, Taekwondo, Soccer	Samuel	Father
Leo	Male	Walks with no help	Swimming, Soccer, Dodgeball	Benedict	Father
Reggie	Male	Walks with hand-held mobility device/uses wheelchair in some environments	Swimming, Basketball, Boxing	Allen	Father
Pete	Male	Walks with hand-held mobility device/uses wheelchair in some environments	Swimming	Talia	Mother
Harry	Male	Walks with hand-held mobility device/uses wheelchair in some environments	Soccer	Nala	Mother
Nathan	Male	Walks with no help	Muay Thai, Wheelchair basketball	Nessie	Mother
Asher	Male	Walks with no help	Basketball, Baseball, Swimming	Shirley and Randall	Mother and Father
Caden	Male	Walks with no help	Basketball, Baseball, Tennis camp	Shirley and Randall	Mother and Father
Unity	Female	Walks with no help	Swimming, Dance	Saaida	Mother
Stephanie	Female	Walks with no help using ankle-foot orthosis	Swimming, Track and field, Wheelchair basketball, Frisbee	Anna	Mother
Joshua	Male	Uses wheelchair in some environments	Swimming	Penelope	Mother
Pablo	Male	Walks with no help	Baseball, Soccer, Basketball, Flag football, Swimming	Babette	Mother
Sadie	Female	Walks with no help	Horseback riding, Wheelchair basketball, Soccer	Kameron	Mother
Vanya	Female	Walks with no help	Horseback riding, Track and field, Frisbee, Soccer, Basketball, Ball hockey	Lara	Mother
aN/A	Male	Walks with no help	Trampoline, Horseback riding, Gymnastics, Basketball, Swimming	Donna	Mother

a *Note*. All listed children and caregivers participated in the study, except for one caregiver whose child did not participate. In that case, limited child demographic details are provided to contextualize the caregiver’s interview.

**Table 4:** Summary of coach demographic characteristics.

Coach Pseudonym	Sex	Job Title	Highest Level of Education	Years of Experience in Current Position	History of Sport Involvement (Coaching, Supporting, etc.)
Kayla	Female	Therapeutic recreation specialist	Undergraduate university degree	6	Wheelchair basketball, Track and field
Claudia	Female	Soccer coach	Postgraduate degree	1	Ambulatory cerebral palsy soccer, Recreational gym-based sports (e.g., Soccer, Basketball)
Amanda	Female	Therapeutic recreation specialist	Undergraduate university degree	9	Sailing, Wheelchair sports, Dragon boating
Fiona	Female	Occupational therapist	Postgraduate degree	9	Dragon boating, Rock climbing, and various camp sports (e.g., Swimming, Horseback riding, Archery, Canoeing)
Kristina	Female	Therapeutic recreation specialist	Undergraduate university degree	9	Wheelchair basketball
Veronica	Female	Coordinator, head coach	Undergraduate university degree	5	Basketball, Soccer, Tennis, Martial arts, Goalball, Seated volleyball, Guided running, Badminton
Francesco	Male	Professor	Postgraduate degree	>10	Soccer
Larry	Male	Coach and mentor	Undergraduate university degree	1	Various camp sports (e.g., Swimming, Canoeing, Kayaking)
Ashley	Female	Marketing director	Postgraduate degree	1	Soccer
Mary Jane	N/A	N/A	N/A	N/A	Boxing
Alexia	Female	Volunteer boxing instructor	Highschool diploma	1	Boxing
Carly	Female	Physical education teacher, boxing coach	Postgraduate degree	3	Softball, Boxing, Soccer, Gym-based sports

### 3.1 Theme 1: Creating avenues for connection

**Subtheme 1: Relationship Building.** Participants viewed time spent by the coach focusing on building a strong coach-participant relationship as a critical component to positive sport participation. Authentic discussions between the coach and child were felt by coaches and caregivers as particularly important for building rapport between coach and child, with an expectation that coaches would take a genuine interest in the children they coach. Similarly, coaches discussed the importance of developing personal connections with children they coached, which was viewed as contributing to a child’s confidence in them as a relatable coach.

“I think from the start getting to know them and building that rapport so that they see that you're not there to tell them what to do, but you're there to learn what it is that they want to do, and be that support to help them get there.” (Kayla, coach, experience working with children with a disability)

Caregivers also emphasized the value of coaches being approachable and accessible to both children and caregivers, listening well, and valuing each child’s opinions.

“They listen to kids, they treat them as people … they maintain that, you know, instructor-student relationship, but at the same time they talk directly to them, they treat them like their opinion is valued … to me the best coaches are those who actually … they’re interested in what the kids are doing; they treat the kids as people … they listen to them.” (Samuel, caregiver of a child with a disability [Anthony])

**Subtheme 2: Social Facilitator.** Outside of the coach-participant relationship, creating opportunities for socialization among children within sport programs was also identified as part of the coaching role. The coach’s presence was perceived by coaches and caregivers as affecting how children interacted with each other and behaved within a sport program; therefore, part of the coach’s role was identified as social mediators of peer-to-peer relationships. Effective coaching strategies that were discussed as enhancing positive peer interaction included pairing, teamwork, shared common experiences, and unstructured (free) time. The individualized needs of children with and without disabilities was highlighted by coaches and caregivers, with an emphasis on allowing children to lead peer-to-peer interactions and for the coach to assist in fostering relationships, when needed, but also acknowledging that these social interactions should not be forced.

“We want them to have a chance to engage with each other through a shared experience, so things like rock wall climbing or team sports you’re doing something together, so you already have a basis, like a common ground to talk about.” (Fiona, coach, experience working with children with a disability)

“Pairing them up and helping them with each other and seeing each other as, you know, working there together, being friends with each other. You’re not going to necessarily force them all to be best friends, but that they’re all, you know, there together as a group and they can help each other and be supportive as well.” (Samuel, caregiver of a child with a disability [Anthony])

### 3.2 Theme 2: An environment focused on child empowerment

**Subtheme 1: Trust and Advocacy.** Children discussed valuing trust and non-judgmental coaching attitudes and sport environments.

“… a coach, to me is like someone who gives you confidence, someone who is just nice, someone who just is a good person to be around, that you feel like you can trust with sports, and someone you feel like can't judge, they won't judge you.” (Stephanie, age 10, child with a disability)

Additionally, coaches viewed themselves as advocates for children with disabilities so that the ownness of self-advocation is not placed on the child. Kayla highlighted that a coach should model inclusion, whilst mitigating exclusion and bullying, to foster a program environment that strives for fairness and avoids favouritism.

“Yeah, I think sometimes when somebody … you can kinda see right away if somebody either isn't able to, or isn't going … maybe they're capable of it, but they don't want to advocate for themselves because they don't want to be put on the spot. So I think in those cases I would step in. Not in, like, such a direct way to embarrass them or make it even worse. But sometimes it's kind of like in a … what's the word I'm looking for? Not role play, but just … just demonstrating what it is that you want to see from those other participants in terms of acceptance and inclusion.” (Kayla, coach, experience working with children with a disability)

**Subtheme 2: Building Resilience and Personal Growth.** Participants indicated preference for a holistic coaching style to support and empower children with an emphasis on building resilience and personal growth. This empowerment to experience success in an inclusive sport environment ultimately was viewed as a tool to bolster transferable confidence, where coaches who had experience with coaching inclusive programs viewed sport participation as an opportunity to highlight the strengths of each child.

“Just providing them with successful opportunities, so making sure that they are successful and enjoying themselves, so that there’s a little more buy-in so that they’ll want to continue doing those types of things” (Kayla, coach, experience working with children with a disability)

Additionally, some caregivers felt that their children would grow more if the coach challenged them in order for them to build resilience. Caregivers also highlighted that coaches should be able to highlight ‘teachable’ moments to further a child’s personal growth.

“I like them to be tough on Nathan because that way he feels like … he feels, I think like, it makes him feel like he's … he's like, more confident, right? He's getting more strength. More strength and confidence that's important to me, and I look for that too in a coach … if the kid's just doing something wrong, I like the coach to like be direct, right? Address the situation right away, and then you can move on.” (Nessie, caregiver of a child with a disability [Nathan])

### 3.3 Theme 3: Autonomy-supportive coaching practices

**Subtheme 1: Leadership Opportunities for the Children.** Coaches and caregivers suggested coaches should provide peer leadership opportunities as part of promoting inclusion and quality sport experiences. This was fostered through coaches promoting self-efficacy and building capacity amongst program participants in facets other than sport skill. Effective coaches were perceived by coaches and caregivers as being able to provide different methods of participation to allow all children, regardless of ability, to receive a valuable leadership experience. Leadership opportunities were not awarded to those with exceptional skill, but rather were used to enable varying perspectives and ideas to permeate the program.

“How I'd do it personally, I'd put them [children with a disability] as a leader actually … They decide … like they make some of the … they make some of the big decisions as a group. And I treat them equitably pretty much, not equally, but equitably. And … yeah, mainly I think it's putting them in a leadership position that gives them that confidence that yes even if you're different, yes, we still want you as a leader because you have a lot to offer ... a different perspective, different ideas.” (Veronica, coach, experience working with children with disabilities)

“Oh I think it's great [youth being leaders for each other]. I think it's great. I think it's really important 'cause it forges relationships, like, in … sometimes if it, even if it's an older age that's leading the younger age then it forges a relationship within that age group that would normally not socialize. And it teaches the kids to be responsible, and it teaches them … Little baby steps to being an adult.” (Lara, caregiver of a child without a disability [Vanya])

**Subtheme 2: Motivation Enhancement.** For participants in this study, empowering children with autonomy often had a significant impact on a child’s motivation to engage in activities. Motivation and engagement were common threads of discussion, often related to opportunities for choice within programming. Equipping children with the capacity to make their own decisions was mentioned as having a positive effect on children’s engagement, as observed by caregivers and coaches. Children reported occasions where they felt respected and comfortable to be themselves.

“It was good ‘cause … they [swim coaches] didn’t push you too much, and if you need to take a break they understood that.” (Stephanie, age 10, child with a disability)

Although caregivers recounted their children’s most positive sport experiences as autonomy-supportive, they also emphasized the differing needs their children possess to facilitate meaningful engagement in group sport settings. This was highlighted through the value of the scaffolding approach, where coaches provide support and motivation for children to participate, but no more than what they individually need.

“You’re trying to remove yourself as much as possible so for the most part you’re trying to build that independence skill. And maybe they need more support up front, but by the time they’ve done it a few times then you kind of step back right? So it’s … I guess when I say scaffolding I guess I just mean supporting as much as you need to, but no more than that.” (Fiona, coach, experience working with children with disabilities)

**Subtheme 3: Goal Pursuit and Attainment.** Goals were deemed a valuable tool in activity engagement and providing a sense of accomplishment upon achievement. Highlighting the importance of an individualized approach to working towards goals, the need to navigate competing priorities of caregiver and child was often mitigated by coaches taking a child-centered approach.

“I do think that goal setting is really important … for them to have something that they can look forward to, you know, it’s like what do they want to get out of it? When Anthony really wanted to do better at, you know, he noticed he wasn’t advancing as far as he wanted in the belts (Taekwondo), he wanted to do more. He had a goal, and that he and his instructor sat down and figured out how they’re going to do it.” (Samuel, parent of a child with a disability [Anthony])

“We always want to meet with them and find out more about them, what their goals are, what their needs are and then we can plan a program that matches that. We’re not really looking for things that would make it so that they can’t participate, but we’re looking for what do we need to put in place so they can.” (Amanda, coach, experience working with children with a disability)

### 3.4 Theme 4: Flexibility and adapting the program to suit everyone

**Subtheme 1: Skill Development and Differentiation.** Coaches, children, and caregivers highlighted skill differentiation as a key element in supporting successful and enjoyable experiences. Meeting individuals where they are at requires commitment to progress, learning, and achievement. Coaches and children indicated that close observation of children was important to differentiate coaching techniques for each child’s needs.

“… a coach’s job is to identify strengths and help somebody use those strengths to improve. I think it's … it's that supportive role that you're there to kinda help navigate and guide.” (Kayla, coach, experience working with children with disabilities)

Coaches require the ability to differentiate skills and identify abilities, so every child is effectively challenged and supported, including preparing appropriate equipment and considering other environmental factors. Essential components to consider when addressing skill differentiation included the importance of enjoyment, as well as selecting appropriate activities and equipment that engage *and* challenge all participants. Without this, children lack equitable opportunities to learn and grow through sports programming.

“We were only ever doing the lower-level things, so everyone was equally good at them. I kinda felt bored sometimes ‘cause we didn’t get to do a lot of fun stuff that I really wanted to do because I was good at it.” (Anthony, age 11, child with a disability)

This quote highlights that if aiming for *equal* participation, programs may unintentionally under-challenge some participants. A tension arose in the data between universal programming approaches, where participants all do the same activity, and individualized approaches that focus on differentiated skill development. Some caregivers preferred uniformity across participants for perceived inclusion, while others emphasized the need for adaptations that allow each child to progress at their own pace.

“You know, like if there’s a kid that has balance issues then, you know, you would alter the game to be played this way so that all the kids are now playing this way as opposed to just the child that has a problem with it, right? It would be nicer if they were able to participate all in the same … like doing a dance class, they would do all the same type of dance as opposed to one or two kids doing a dance in an alternate way.” (Kameron, caregiver of a child with a disability [Sadie]).

These perspectives reflect different understandings of fairness and inclusion, whether that means equal treatment through uniform programming, or equitable treatment through differentiated approaches that meet individual needs.

**Subtheme 2: Thinking Outside the Box.** The skill or characteristic of creativity, in coaches, is paramount. Participants described creativity as an intangible coaching skill, apparent in a coach’s ability to perceive and adapt based on an assessment of individual needs, goals, and interests, which often requires ‘outside of the box’ thinking. A strong relationship with program participants as well as an understanding of goals and interests provides a basis for matching activities accordingly. Coaches shared that creativity is not always intuitive; however, children can be effective sources for creative ideas and inspiration for program variation.

“[A good coach is] Probably someone who’s a creative outside of the box thinker that can sorta do things a little differently and not, you know, not one of those people that’s you know, those teachers that are like we need to learn X, Y, Z and that’s it, there is no W. Right? I think she needs someone who is, you know, willing to adapt and … change, and someone who’s willing to completely change a program as well and do things to make it better for all the kids.” (Kameron, caregiver of a child with a disability [Sadie])

“I think the role of the facilitator is to assess what supports are needed in that situation in that moment when it doesn’t work. Maybe you do need to stop an activity and support [children] in a different way, so I think all of those are, like, in the moment assessment skills that you need to have.” (Fiona, coach, experience working with children with a disability)

## 4 Discussion

Through interviews with children, caregivers, and coaches, this study sought to understand the role coaches play in optimizing the experiences of children who participate in recreational group sport programs that include children with and without disabilities. Findings reflected four interrelated themes: creating avenues for connection, fostering environments focused on child empowerment, adopting autonomy-supportive coaching practices, and ensuring flexibility to adapt programming to suit everyone. Across participant groups, there was a shared emphasis on the importance of inclusive coaching practices that prioritize relationship-building, respect children’s strengths, and promote both skill development and enjoyment. Our findings indicate the need for coach proficiency in sport- and non-sport-related skills to ensure meaningful participation experiences.

The developmental model of sport participation posits that an emphasis must be placed on dedicated training of coaches to foster quality participation experiences at an early age to reduce the risk of sport dropout in children during or after the sampling phase [4]. Quality sport participation for individuals with disabilities has been conceptualized within the quality parasport participation framework in the context of parasports [12] as well as other physical activity settings (e.g., exercise programs; [39]). This framework stipulates that, with the proper environmental conditions, quality sport experiences occur by satisfying one or more of six experiential elements: autonomy, belongingness, mastery, challenge, engagement, and meaning [12]. Repeated exposure to these experiential elements is hypothesized to contribute to quality sport participation – that is feeling satisfied with and pleasure in one’s sport pursuits, and that one’s sport participation enables the achievement of meaningful outcomes [12].

While not initially positioned within the quality parasport participation framework, several findings from this study align with the framework’s experiential elements. For example, many of the caregiver participants described how coaches who focus on fostering their child's autonomy and motivation contribute to more positive experiences in sport. Autonomy-supportive coaching styles can facilitate creation of leadership opportunities for children with and without disabilities [40]. This is crucial within programming that includes children with disabilities, as historically, coaches have resorted to providing leadership positions to children with higher skill levels, which in many cases, has resulted in children with disabilities not being selected for leadership roles [27]. Providing leadership roles only to individuals without disabilities in an integrated program setting can create a detrimental cycle where individuals with less sport-specific skills are not provided with equal opportunities to grow and develop leadership skills [41]. Adopting an autonomy-supportive coaching style would, therefore, allow children to self-select leadership opportunities throughout practice.

In addition to autonomy-support, coaches, children, and caregivers emphasized the importance of coaches facilitating social interactions, as well as the development of other non-sport-specific skills (e.g., self-confidence) that could then be utilized within the program setting. Sport programming in inclusive settings provides opportunities for children with diverse abilities to interact with, and learn from, each other [42]. Being part of an inclusive group can also reduce stigma and stereotyping behaviours towards children with disabilities [43]. Coaches play a key role in fostering belongingness in sport environments for children [12,44]. However, participants highlighted that friendships should not be forced. Sport provides children with a shared (relatable) experience, although not all children will form strong and lasting friendships with each other. Coaches, therefore, should provide children with the tools to interact with one another, while allowing independence in how these interactions occur [45]. While leadership opportunities play a role in fostering social interactions, the role of a leader should not be the sole factor in determining the motivation behind social interactions. If a coach provides opportunities for children to develop social skills while in sport settings, children will be able to practice these skills and foster a sense of belongingness within sport [46].

Caregivers in this study described varying degrees of involvement in sport-related decision-making, reflecting a broader spectrum of engagement that ranged from being regularly consulted by coaches to having limited communication about their child’s sport experiences. These differences demonstrate the complex and context-dependent nature of caregiver-coach relationships in inclusive sport programs. In some cases, caregivers were brought into conversations around goal-setting and program adaptations, while in others, the coach-child dyad remained the primary focus. This variability is consistent with the broader principles of family-centered care in healthcare, which emphasize the importance of caregiver knowledge, shared decision-making, and family-friendly environments [[47], [48], [49]]. However, unlike healthcare, inclusive sport programs tend to offer more fluid and informal roles for caregivers, with involvement shaped by organizational structure, available resources, and family preference. For instance, in resource-constrained environments, caregivers may take on more active roles in program delivery, even supporting elements of coaching [29]. While such roles were not explicitly reported by participants in this study, caregivers did describe contributing valuable information about their child’s needs, which aligns with previous findings on caregiver expertise in adapted sport contexts [50,51]. These findings suggest that intentional efforts to build relationships with caregivers can enhance inclusive coaching practices. Future research may explore how sport organizations can equip coaches to meaningfully engage caregivers while respecting child autonomy and promoting inclusive program delivery.

As indicated by children, caregivers, and coaches within this study, challenging children with and without disabilities in sport settings is necessary for developing competence and confidence. Importantly, our findings revealed tension between two approaches to inclusive sport-delivery, including uniform programming versus individualized differentiation. While some caregivers emphasized the value of all children participating in the same way to promote a sense of inclusion and togetherness, others, including children themselves, expressed frustration when programming lacked sufficient challenge or individualization. These divergent perspectives reflect ongoing debates in inclusive education and sport about whether fairness means treating everyone the same or equitably responding to diverse needs [52]. Coaches must, therefore, balance consistency across the group with flexibility to differentiate tasks so all children can experience challenge.

Additionally, some caregivers expressed wanting coaches to challenge their child, because they believed that the child would not motivate themselves. While challenge is one of the experiential elements contributing to quality participation experiences in sport, it is important for coaches (and caregivers) to differentiate between challenge and being ‘tough’, as the latter may contribute to sport drop out. While a caregiver may feel ‘tough love’ contributes to resilience, motivation can be provided in positive ways (e.g., autonomy-supportive coaching) that also contribute to building resilience. In contrast, coaches and individuals in coach-adjacent roles (e.g., teachers) often assume children with disabilities are less competent than their peers without disabilities and should not be challenged [53]. As a result, children with disabilities are more often side-lined in the role of ‘helper’ and provided with fewer opportunities for skill development [24]. Coaches should, therefore, provide all children with the opportunity to build self-confidence, but also find activities where they feel sufficiently challenged [12]. Learning each child’s limits and ensuring activities provide appropriate challenge are critical for the child to develop skill mastery and feel competent and confident due to this skill acquisition [54].

### 4.1 Study limitations and future directions

While the study findings shed insight into coach knowledge, skills, and behaviours that may optimize the quality of sport experiences for children in inclusive group sport programs, several limitations must be acknowledged. First, the study included a relatively small number of children with disabilities, all of whom were between the ages of 10–13 and participated in recreational-level sport. As such, the findings may not reflect the experiences of children at different developmental stages, particularly those under the age of 10. Further research is needed to explore sport sampling experiences across a broader age range.

The sample included children with physical and neurodevelopmental impairments; however, not all types or severities of these impairments were represented. Because participation required the ability to engage in a semi-structured interview, the sample was likely skewed toward children with higher levels of cognitive, verbal communicative, and behavioural functioning. This limits the generalizability of findings to children with more significant cognitive or communication support needs. Additionally, children with sensory impairments were not included, indicating an important gap for future research.

Participants in this study were drawn from both integrated and segregated sport settings. In the Canadian context, recreation-level sport programming for children with disabilities varies substantially by municipality; in some communities, segregated programs are unavailable due to population size and limited resources. Including both types of settings helped capture a broader range of lived experiences; however, future work might explore how the findings translate across contexts with different structural or philosophical approaches to inclusion.

Finally, while the coach participants represented a range of educational backgrounds, professional roles, and sport types, the sample may not be representative of the breadth of coaching contexts across Canada. Most had experience in community-based or therapeutic recreation programs with a concentration in adapted or inclusive sports. Coaches also varied in experience, with several having fewer than three years in their current roles. Although this diversity offered valuable insight into inclusive recreational coaching, the perspectives of coaches in more competitive sport settings were less represented, and experience coaching certain sports (e.g., hockey) was absent. Future research should aim to include a broader cross-section of coaches across different competitive levels, organizational structures, and geographic locations to explore whether the findings apply across diverse sport and physical activity environments.

## 5 Conclusion

Coaches play a pivotal role in shaping the quality experiences of children in sport programming. Our findings indicate the need for coaches to demonstrate high-level skills to effectively support children with diverse abilities. These skills include: the ability to be creative and strategic with skill differentiation, adapting to the immediate needs of each child, supporting children’s autonomy, and providing progressive feedback that balances positive reinforcement with challenges. This is an important first step towards ensuring quality participation of children with disabilities in sport.

## Declaration of competing interest

The authors declare the following financial interests/personal relationships, which may be considered as potential competing interests: Kelly Arbour-Nicitopoulos reports financial support was provided by Government of Canada Social Sciences and Humanities Research Council. Roxy O’Rourke reports a relationship with Government of Canada Social Sciences and Humanities Research Council that includes: funding grants. If there are other authors, they declare that they have no known competing financial interests or personal relationships that could have appeared to influence the work reported in this paper.
