# Supplementary material for: Developing disability inclusive coaching principles within community recreational sport programming for children
Source: JSAMS Plus. 2025 Jul 9;6:100109. doi: 10.1016/j.jsampl.2025.100109 (PMC13008455; doi:10.1016/j.jsampl.2025.100109)
Supplement: Multimedia component 1 [file mmc1.docx]

**Supplementary Material**

**Child Interview Guide**

“Thank you for talking to me today. There are a couple words that I will use a lot in this discussion, and I want to make sure you understand what I mean by them before we get started. The first is ‘inclusive physical activity’. Physical activity includes any play, game, sport or exercise that gets you moving, breathing harder, and your heart beating faster. Inclusive physical activity is an activity that involves everyone, no matter what their ability is (e.g. children who use a wheelchair or walker, or children who take longer understanding what to do). Everyone is given the chance to discover what they are really good at, what activities they like to do, and learn new sports and fitness skills. Do you have any questions so far? We would like to hear about your experiences and ideas about participating in inclusive physical activity programs. I would like to record our conversation so that I don’t forget anything you say. We won’t tell anyone what you said who is not part of this study. No one else will know you took part.”

***Note:* Questions are just a guide and will be adapted depending upon the child’s development and cognitive ability.**

Can you tell me a little bit about yourself? What types of activities do you like to do? Where do you usually do them and who do you usually do them with? Prompts: alone, friends, family, coaches

Have you ever participated in a physical activity program that involved kids of all physical and intellectual abilities? If so, can you tell me about your experiences in the program(s)? Prompts: What did you enjoy or what didn’t you enjoy about those experiences? What did people do in this program that made it open to all abilities? (If no experience in inclusive physical activity programs, ask about experience in inclusive non-physical activity programs)

Would you like to have more experiences with inclusive physical activity programs? Why or why not? What would you expect to get out of taking part in these kinds of programs? How could these programs be made to be fun for you?

Can you tell me about a time that you did not feel included in a physical activity program? Did this keep you from participating? Why or why not? What did people do in this program that made it *not* inclusive? How would you improve this program to make it more inclusive?

What helps motivate you to do physical activity? Prompts: Social support, physical benefits, emotional benefits, etc.

“We want to know how important you think it is to have someone who can be like a coach to help you participate. We want to know what you would imagine this person would be like and what they would do to help you. A coach could be a lot of different things—a teacher, an instructor, a personal trainer, someone who does the physical activity with you, someone you can go to for questions or advice, etc. What we really want to know is how you would describe this person.”

Can you tell me about the relationships you have developed OR would develop in inclusive physical activity programs? Prompts: With kids or adults?

If you had someone there to help you participate in inclusive physical activity programs, what would that person do? Prompts: Help reach individualized goals? Modify activities? Observe and give feedback (e.g. social, physical)? How?

How would you want to interact with this person? Prompts: One-on-one, in the program, at home, in the community, via technology. How often would you want to see this person?

How would you feel about having someone there to help you to participate and/or feel included?  How important is this to you?

What would be important for the ‘coach’ to know so they could be the most helpful to you? Prompts: Understanding of disability or health, knowledgeable about physical activity or social skills?

What do you imagine this person would be like? Prompts: Top three characteristics

Do you feel that having a ‘coach’ would change how you interact with your friends or teammates? Why or why not? How?

What words would you use to describe this person? Prompts: Coach, personal trainer, instructor, etc. *(Ask this question earlier if fitting)*

**Parent Interview Guide**

“Thank you for talking to me today. There are a couple terms that I will use a lot in this discussion, and I want to make sure you understand what I mean by them before we get started. The first is ‘inclusive physical activity’. Physical activity includes any play, game, sport or exercise that gets your child moving, breathing harder, and his/her heart beating faster. Inclusive physical activity is an activity that involves kids of all physical and intellectual abilities (e.g. children who use a wheelchair or walker, or children who take longer understanding what to do). In an inclusive physical activity program, everyone in the group is able to participate fully and in meaningful way together. Everyone is given the chance to discover what they are really good at, what activities they like to do, and learn new sports and fitness skills. And the last term is ‘coach’. A coach could be a lot of different things—a teacher, an instructor, a personal trainer, someone who does physical activity with your child, someone you can go to for questions or advice, etc. What we really want to know is how you would describe this person and their role. Do you have any questions so far? We also would like to hear about your experiences and ideas about your child participating in inclusive physical activity programs. We want to know how important you think it is to have someone who can be like a coach to help your child to participate. Finally, we want to know what you would imagine this person would be like and what they would do to help your child. I would like to record our conversation so that I don’t forget anything you say. We won’t tell anyone what you said who is not part of this study. No one else will know you took part.”

Can you tell me a little bit about your child? What types of activities do you encourage your child to join? How does your child feel about participating in these activities? Where does he/she usually do them and who does he/she usually do them with? Prompts: alone, friends, family, coaches.

Has your child ever participated in a physical activity program that involved kids of all physical and intellectual abilities? If so, can you tell me about these program(s)? Prompts: What did your child enjoy or what didn’t he/she enjoy about those experiences? What did people do in this program that made it open to all abilities? (If no experience in inclusive physical activity programs, ask about experience in inclusive non-physical activity programs)

Would you like your child to have more experiences with inclusive physical activity programs? Why or why not? What would you expect your child to get out of taking part in this kind of program? Prompts: Three things you would like your child to learn in these programs.

Can you tell me about a time that you felt your child was not included in a physical activity program? Did this keep your child from participating? Why or why not? What did people do in this program that made it not inclusive? How would you improve this program to make it more inclusive?

In your opinion, what helps motivate your child to do physical activity? Prompts: Social support, physical benefits, emotional benefits, etc. How do you try to motivate your child to do physical activity?

Can you tell me about the relationships your child has developed OR relationships you would like your child to develop in inclusive physical activity programs? Prompts: With kids or adults (e.g. staff)? Quality of friendships?

If there was someone to help your child participate in inclusive physical activity programs, what would their purpose be? Prompts: Help child achieve individualized goals? Modify activities? Observe and give feedback (e.g. social, physical)? How?

How would you want your child to interact with this person? Prompts: One-on-one, in the program, at home, in the community, via technology. How often do you think it would be reasonable for these ‘coaching’ sessions to occur?

How would you feel about having someone there to help your child to participate and/or feel included? How important is this to you?

What would be important for the ‘coach’ to know so they could be the most helpful to your child? Prompts: Understanding of disability or health, knowledgeable about physical activity or social skills?

What do you imagine this person would be like? Prompts: Top three characteristics

Do you feel that having a ‘coach’ would change how your child interacts with his/her friends or teammates in the program? Why or why not? How?

What words would you use to describe this person? Prompts: Coach, personal trainer, instructor, etc. *(Ask this question earlier if fitting)*

**Coach Interview Guide**

“Thank you for talking to me today. There are a couple terms that I will use a lot in this discussion, and I want to make sure you understand what I mean by them before we get started. The first is ‘inclusive physical activity’. Physical activity includes any play, game, sport or exercise that gets children moving, breathing harder, and their hearts beating faster. Inclusive physical activity is an activity that involves kids of all physical and intellectual abilities. In an inclusive physical activity program, everyone in the group is able to participate fully and in meaningful way together. Everyone is given the chance to discover what they are really good at, what activities they like to do, and learn new sports and fitness skills. And the last term is ‘coach’. A coach could be a lot of different things—a teacher, an instructor, a personal trainer, someone who does the physical activity alongside the children, someone who children can go to with questions or for advice, etc. What we really want to know is how you would describe this person and their role. Do you have any questions so far? We also would like to hear about your experiences and ideas about leading inclusive physical activity programs. We want to know how important you think it is to have someone who can be like a coach to help children of all abilities participate. Finally, we want to know what you would imagine your role would be. I would like to record our conversation so that I don’t forget anything you say. We won’t tell anyone what you said who is not part of this study. No one else will know you took part.”

Can you tell me a little bit about yourself and your experience as a ‘coach’? (Interviewer will adapt terminology to participant’s vocabulary)

Have you ever ‘coached’ an inclusive physical activity program (one that includes children of all abilities)? If so, can you tell me about these program(s)? Prompts: What did you or other people do in this program that made it inclusive or open to all abilities? (If no experience in inclusive physical activity programs, ask about experience in non-inclusive pa programs and perceptions of inclusive pa programs) What are some of the challenges or barriers that get in the way of inclusive physical activity programming?

Would you like to have more experiences with inclusive physical activity programs? Why or why not? What would you expect children to get out of taking part in this kind of program? Prompts: Three things you feel children could learn in these programs.

Can you tell me about a time that you felt a child was not included in a physical activity program? What did you do to help mitigate this? What did people do in this program that made it not inclusive? Prompts: yourself, other staff, children and their peers. How would you improve this program to make it more inclusive?

How do you try to help children be motivated to participate in physical activity? Prompts: Social support, physical benefits, emotional benefits, etc.

Can you tell me about the relationships you have observed in inclusive physical activity programs? Prompts: Between children or with ‘coach’/other staff? Quality of friendships?

How do you perceive your role in helping children participate in inclusive physical activity programs? Prompts: Help child achieve individualized goals? Modify activities? Observe and give feedback (e.g. social, physical)? How?

How would you want to interact with children in inclusive physical activity programs? Prompts: One-on-one, in the program, at home, in the community, via technology. How often do you think it would be reasonable for these ‘coaching’ sessions to occur?

How do you imagine the role of the parent in the ‘coaching’ process? Prompts: Would the parent be involved in regular sessions, an onlooker to the sessions, separate progress meetings, not at all, etc.

How important do you feel it is that children have a ‘coach’ in inclusive physical activity programs?

What would be important for you to know so they could be the most helpful to your child? Prompts: Understanding of disability or health, knowledgeable about physical activity or social skills? What experience and/or training do you feel you would need to be able to be a ‘coach’ in inclusive physical activity programs?

Do you feel that having a ‘coach’ would change how children interact with each other in inclusive physical activity programs? Why or why not? How?

What words would you use to describe this role? Prompts: Coach, personal trainer, instructor, etc. *(Ask this question earlier if fitting)*
